# Myosin 1b Regulates Amino Acid Transport by Associating Transporters with the Apical Plasma Membrane of Kidney Cells

**DOI:** 10.1371/journal.pone.0138012

**Published:** 2015-09-11

**Authors:** Shigeru Komaba, Lynne M. Coluccio

**Affiliations:** Department of Physiology & Biophysics, Boston University School of Medicine, Boston, Massachusetts, United States of America; National Cancer Institute, UNITED STATES

## Abstract

Amino acid transporters (AATers) in the brush border of the apical plasma membrane (APM) of renal proximal tubule (PT) cells mediate amino acid transport (AAT). We found that the membrane-associated class I myosin myosin 1b (Myo1b) localized at the apical brush border membrane of PTs. In opossum kidney (OK) 3B/2 epithelial cells, which are derived from PTs, expressed rat Myo1b-GFP colocalized in patched microvilli with expressed mouse V5-tagged SIT1 (SIT1-V5), which mediates neutral amino acid transport in OK cells. Lentivirus-mediated delivery of opossum Myo1b-specific shRNA resulted in knockdown (kd) of Myo1b expression, less SIT1-V5 at the APM as determined by localization studies, and a decrease in neutral AAT as determined by radioactive uptake assays. Myo1b kd had no effect on Pi transport or noticeable change in microvilli structure as determined by rhodamine phalloidin staining. The studies are the first to define a physiological role for Myo1b, that of regulating renal AAT by modulating the association of AATers with the APM.

## Introduction

In the kidneys, amino acids in the blood are filtered at the glomeruli and absorbed through the PTs of nephrons [1–3]. PT epithelial cells contain on their apical surface a brush border of actin-filled microvilli that increase the surface area and are required for maintenance of cell polarity; the brush border is the site of amino acid reabsorption [4]. Multiple transporter systems mediate the uptake of single amino acids across the luminal membrane [1–3,5]. The broad scope B^0^ transport system is responsible for the Na^+^-dependent uptake of neutral amino acids in both kidney and intestine [6–8]. The major apical AATer is B^0^AT1 (*SLC6A19*), which transports all neutral amino acids to different degrees with preference for leucine, isoleucine, valine and methionine [1,6]. Mutations in B^0^AT1 are associated with Hartnup disorder, an aminoaciduria associated with a skin rash and cerebellar ataxia [8,9]. The B^0^AT1-related transporter SIT1 is a B^0^-type high-affinity L-imino acid (proline and related compounds including hydroxyproline, betaine, N- methylaminoisobutyric acid and pipecolic acid) transporter expressed in epithelial cells of intestine and kidney [10,11]. SIT1 is the predominant proline transporter in OK 3B/2 cells [12,13], although the sodium-coupled neutral amino acid transporter 2 (SNAT2) mediates proline transport during amino acid deprivation [13]. In OK 3B/2 cells, SIT1 is also a low-affinity Na^+^-dependent neutral AATer, which may also describe its function in humans [4,10,12].

Localization studies in murine kidney indicate that B^0^AT1 is expressed mainly in the early part of the PT, whereas SIT1 is expressed along all segments of the PT [4]. The localization of both transporters at the luminal brush border depends on the membrane protein collectrin (*Tmem27*), which shares sequence similarity with the non-catalytic extracellular, transmembrane and cytosolic domains of angiotensin converting enzyme 2 (ACE2) [14]. Mice lacking collectrin have fewer AATers including B^0^AT1 and SIT1 at their renal APM and exhibit severe leakage of nearly all amino acids [15,16]. Collectrin associates with Soluble NSF Attachment Protein Receptor (SNARE) complexes, which mediate vesicular fusion events, suggesting that collectrin mediates the intracellular trafficking of AATers and their fusion with the APM [17,18].

The actin cytoskeleton interacts with membrane channels, receptors and transporters either directly or indirectly to support their intracellular trafficking [19]. Actin-binding proteins regulate the actin cytoskeleton by mediating actin assembly, structure and function [20]. In particular, myosins constitute a superfamily of actin-associated molecular motor proteins that convert the energy from ATP hydrolysis into movement and force [21,22]. Their functions include intracellular transport of membrane-bound vesicles, regulation of membrane tension, anchoring of membrane-bound organelles, and tethering of membrane-bound proteins. Twelve classes of myosins are expressed in humans [23]. The membrane-associated class I myosins are the most diverse with 8 different subclasses, Myo1a-h [24]. The best known is Myo1a, whose expression is restricted to intestine [25], where it links the core bundle of actin filaments in microvilli to the microvillar membrane [26,27]. Myo1a binds and localizes sucrase-isomaltase [28], and it supports the movement of membrane along microvillar actin bundles resulting in the release of vesicles from microvillar tips [29]. *Myo1a*-null mice have fewer CFTR channels and reduced ion transport [30].

Myo1b, a close relative of Myo1a, is found in membrane protrusions including lamellipodia, filopodia and ruffles, and is associated with intracellular organelles [31–37]. Myo1b binds the phospholipids phosphatidylinositol 4,5-bisphosphate (PIP_2_) and phosphatidylinositol 3,4,5-trisphosphate (PIP_3_) with high affinity through a putative pleckstrin homology (PH) domain in the carboxyl-terminal tail domain [33]. Biochemical studies with tissue-purified and expressed proteins show that the interaction of Myo1b with actin is kinetically slow and biphasic [38]; moreover, single molecules of Myo1b interact with actin in two sub-steps, which may be coupled to Pi followed by ADP release [39]. These observations and studies showing that the rate of detachment of single Myo1b molecules from actin decreases significantly under tension [40] suggest that the interaction of Myo1b with actin is strain dependent: release of strain allows ADP to dissociate from Myo1b in order for Myo1b to complete its power stroke [38,41]. Together, the available data suggest that Myo1b holds actin filaments in place at membranes [41]. This is consistent with studies showing that cortical tension increases in cells expressing myosins I including Myo1b [42]. Other studies show that expression of Myo1b affects the distribution of endocytotic compartments suggesting a role in endocytosis [36], and Myo1b couples actin assembly to organelles and controls membrane remodeling at the trans-Golgi network [43].

To date, no physiological role for Myo1b has been described. We found that Myo1b localized at the apical brush border of renal PTs and in the patched microvilli on the APM of OK 3B/2 cells where it colocalized with SIT1-V5. In OK 3B/2 cells expressing Myo1b-specific shRNA, which reduces the amount of expressed Myo1b, less SIT1-V5 localized at the APM. Furthermore, AAT was inhibited in Myo1b-kd cells as determined by transport assays with radiolabeled isoleucine. In contrast, Myo1b kd had no effect on the gross structure of the apical microvilli or inorganic phosphate transport. The data support a model in which Myo1b modulates renal AAT by tethering AATers to the APM.

## Materials and Methods

### Partial cloning of OK Myo1b cDNA

Total RNA was purified from OK 3B/2 cells using an RNeasy mini kit (QIAGEN). cDNA was synthesized with oligo dT primers and used as a template. Opossum Myo1b was amplified with the forward primer ATGCTGGATGARGAGTGCC and the reverse primer CTTGGTGGCCATTTCAATCAGGTG and cloned into pCR 2.1 TOPO (Life Technologies/Invitrogen, Grand Island, NY, USA). Three clones of different sizes were selected and partially sequenced. Class I myosins have an N-terminal motor domain consisting of the ATP-binding site and actin-binding site; a light-chain binding domain with one or more stretches of ~29 amino acids with the consensus sequence IQXXXRGXXXR to which calmodulin binds (referred to as IQ domains) [44]; and a C-terminal tail region that contains a membrane-binding domain [24]. We determined that the clones represent the C-terminus of the motor domain, the IQ region, and about half of the tail region including the putative PH homology domain for the 4IQ, 5IQ and 6IQ isoforms. Thus, each of the three Myo1b isoforms known to exist is expressed in OK cells. The 4 IQ clone, 1662 bp in size, was completely sequenced and used to design shRNA.

### Plasmid construction

To produce pLenti-Myo1b-GFP, cDNA encoding full-length rat Myo1b-GFP excised from pMyo1b-eGFP-N1 with NheI and XhoI was subcloned into the XbaI and SalI sites of pLenti-GFP-Neo (plasmid 17447, Addgene, Cambridge, MA, USA; [45]). For collectrin expression, the GFP cassette of pLenti-CMV-GFP-Hygro (Addgene plasmid 17446; [45]) was excised with BamHI and SalI and replaced with the cDNA of human collectrin (*Tmem27*) prepared by PCR from Tmem27-pCMV6-XL5 (SC122895; Origene, Rockville, MD) with the appropriate restriction sites. As a control, pLenti-CMV-Hygro was prepared and used.

To produce SIT1-V5, the cDNA of mouse SIT1 (aka XT3s1), prepared from mouse kidney RNA, was subcloned into pLenti-CMV-Blast (Addgene plasmid 17486; [45]) using BamH1 and XbaI. Lentiviral vectors for the expression of Myo1b-specific shRNA were prepared using the lentiviral vector pLKO.1 puro (Addgene plasmid 8453; [46]) linearized with the restriction enzymes EcoRI and AgeI and ligated with the following sets of oligonucleotides: #243 (CCG GAG CCA TTC TCT AAT AAA GGC TCT CGA GAG CCT TTA TTA GAG AAT GGC TTT TTT G; AAT TCA AAA AAG CCA TTC TCT AAT AAA GGC TCT CGA GAG CCT TTA TTA GAG AAT GGC T); #628 (CCG GAA GAA TAT TCC TTT GGT AGA TCT CGA GAT CTA CCA AAG GAA TAT TCT TTT TTT G; AATTCAAAAAAAGAA TAT TCC TTT GGT AGA TCT CGA GAT CTA CCA AAG GAA TAT TCT T); and #891 (CCG GAG GAT GGA AGG CTC GAA AGA TCT CGA GAT CTT TCG AGC CTT CCA TCC TTT TTT G; AAT TCA AAA AAG GAT GGA AGG CTC GAA AGA TCT CGA GAT CTT TCG AGC CTT CCA TCC T). In each case, the target site is in the motor domain, which is common to the 4IQ, 5IQ and 6IQ isoforms. Vectors were purified using a Nucleobond Xtra Midi kit (Macherey-Nagel, Düren, Germany) followed by ethanol precipitation, then subjected to automated sequencing.

### Lentivirus production

For lentivirus production, plasmid coding for Myo1b-GFP, Myo1b-specific shRNA, collectrin, or the transporter SIT1-V5 was transiently transfected along with the packaging vector psPAX2 and the envelope vector pMD2.G into HEK 293T cells grown in 6-well plates with Polyethylenimine Max (Polysciences, Inc., Warrington, PA, USA) [47]. The medium was collected 2–6 d later and used to infect cells with no further processing.

### Cell culture and transfection

OK cells are an established cell line from the kidney of an adult American opossum [48]. OK clone 3B/2 cells [49] were the kind gift of Dr. N. Hernando (Zurich, Switzerland). OK 3B/2 cells were cultured in Dulbecco's Modified Eagle's Medium (DMEM)/Ham’s F-12 medium (1:1) supplemented with 10% FBS as previously described, however with no antibiotic [12,50]. HEK 293T cells were grown in DMEM with 10% FBS. To infect OK 3B/2 cells with Myo1b-specific shRNA, 1 ml lentivirus, 1 ml medium, and polybrene to a final concentration of 8 μg/ml were added to cells plated in 6-well plates. Stable shRNA cell lines (bulk) were prepared by selection in 2 μg/ml puromycin. In the case of shRNA, we predict that the rate of transfection is close to or equal to 100% because the number of transfected cells that die during selection is not different from that found in untreated (i.e., not exposed to puromycin) control cultures indicating that all the cells exposed to lentivirus have become infected and have thus acquired antibiotic resistance.

To transiently transfect the stable Myo1b-kd cell lines with collectrin and transporter, 0.25 ml collectrin or empty lentivirus, 0.5 ml transporter lentivirus, 0.25 ml medium and polybrene to a final concentration of 8 μg/ml were added to the Myo1b-kd cells plated in 12-well plates coated with Type 1 collagen (BD Biosciences, Bedford, MA, USA). Cells were assayed after 3 d for the effect of Myo1b kd on either transporter localization using indirect immunofluorescence microscopy or AAT using radioactive transport assays.

### Localization studies

Paraffin sections of mouse kidney were purchased from IHC WORLD, LLC (Woodstock, MD 21163). The studies were approved by the Animal Care and Use Committee and follow the guidelines established by the Panel on Euthanasia of AVMA. Following deparaffinization with xylene and rehydration in an ethanol series, the sections were subjected to antigen retrieval consisting of boiling in 10 mM sodium citrate, pH 6.0 for 5 minutes. Next, sections were permeabilized with 0.1% Triton-X-100 in PBS for 10 min, then blocked with 5% goat serum and 2.5% BSA before incubation with the appropriate primary or isotypic antibodies in PBS with 0.1% saponin, 5% goat serum and 2.5% BSA. After washing, the sections were treated with secondary antibodies (Alexa Fluor 488- and/or Alexa Fluor 594-conjugated goat anti-mouse or goat anti-rabbit), fluorescein-labeled *Lotus tetragonolobus* lectin to label PTs (Vector Laboratories, Burlingame, CA, USA), and Hoechst stain to label nuclei.

Similarly, for localization studies in cultured cells, following transfection, OK cells grown on collagen I-coated coverslips in 12-well plates were fixed in 4% formaldehyde, and permeabilized and blocked for 1 h in 0.1% saponin, 5% goat serum and 2.5% BSA in PBS, then incubated with the appropriate primary antibodies, secondary antibodies and/or rhodamine-labeled phalloidin. Sections and coverslips mounted on glass slides were viewed with a Leica TCS SP5 AOBS 405 UV spectral confocal microscope (Leica, Sohms, Germany). Images were analyzed with Leica advanced fluorescence imaging software and Adobe Photoshop.

To quantitate the percentage of transfected OK cells expressing SIT1-V5 at the APM in control and Myo1b-kd cells, cells were stained with rhodamine phalloidin. Fluorescent images of random areas were taken with a Nikon Eclipse E800 fluorescence microscope (Nikon, Japan). The cells were scored based on whether they expressed SIT1-V5 (i.e., *green*) and whether the localization was cytoplasmic or coincident with that of apical microvilli as determined by colocalization with rhodamine phalloidin (*red*). In each case, 168–465 cells were counted and analyzed.

### Antibodies

Rabbit polyclonal anti-human MYO1B antibodies (HPA013607), mouse monoclonal anti-α-tubulin antibodies (T5168), mouse monoclonal anti-β-actin antibodies (A5441) and normal rabbit IgG were obtained from Sigma-Aldrich (St. Louis, MO, USA). V5 mouse monoclonal antibody (R960-25) and Alexa Fluor 488- or 594-conjugated goat anti-rabbit or goat anti-mouse secondary antibodies were obtained from Molecular Probes/Life Technologies.

### Transport Assays

The amount of [^3^H]isoleucine transported into OK 3B/2 cells was determined for control and Myo1b-kd cells using a modification of a previously described method [12]. Monolayers were washed three times in Buffer A (10 mM HEPES-Tris, pH 7.4; 1 mM CaCl_2_, 5 mM KCl, 1 mM MgCl_2_, 137 mM NaCl, 10 mM glucose) containing 2 mM K_2_PO_4_. Next, the cells were treated with 500 μl AAT Assay Buffer consisting of Buffer A with 5 mM isoleucine and 5 μCi/ml [^3^H]isoleucine, and then incubated at 37°C for 10 min. The buffer was removed, and the cells were washed three times in ice-cold Buffer A and then lysed in 500 μl lysis buffer (0.2% SDS). The lysates were incubated at room temperature for 10 min, then transferred to a 1.5 ml microfuge tube and placed on a Tomy MT-360 microtube mixer (Tomy Seiko, Tokyo, Japan) at speed 10 for 10 min. A 400-μl aliquot was removed and added to 5 ml EcoscintA LS-273 (National Diagnostics, Atlanta, GA, USA), then counted by liquid scintillation in a Packard Bioscience (Meriden, CT, USA) Tri-Carb 2900TR liquid scintillation counter. The data were expressed as nmoles/10 min/dish.

To measure phosphate transport, OK 3B/2 cells grown to confluency in 12-well plates were washed 3 times with Buffer A at 37°C. To each well, 500 μl Buffer A containing 0.1 mM phosphate and 1 μCi/ml ^32^PO_4_ was added to each well and incubated at 37°C for 10 min. The buffer was removed, and the cells were washed 3 times with ice-cold Buffer A, then the cells were lysed in 0.2% SDS for 10 min at room temperature. From each well, the cell lysate was removed to a 1.5 ml microfuge tube and placed on the Toby mixer for 10 min, then 100 μl was counted in 5 ml EcoscintA LS-273.

### Statistics

Data from multiple independent experiments were expressed as means ± s. e., and were analyzed by one-way ANOVA followed by Student's t-test. Data from multiple samples were expressed as average ± s.d. For internal pair-wise comparisons, ANOVA post-hoc tests were performed.

## Results

### Myo1b localized to the microvillar membrane of renal PT cells with AATers

**Fig 1:**
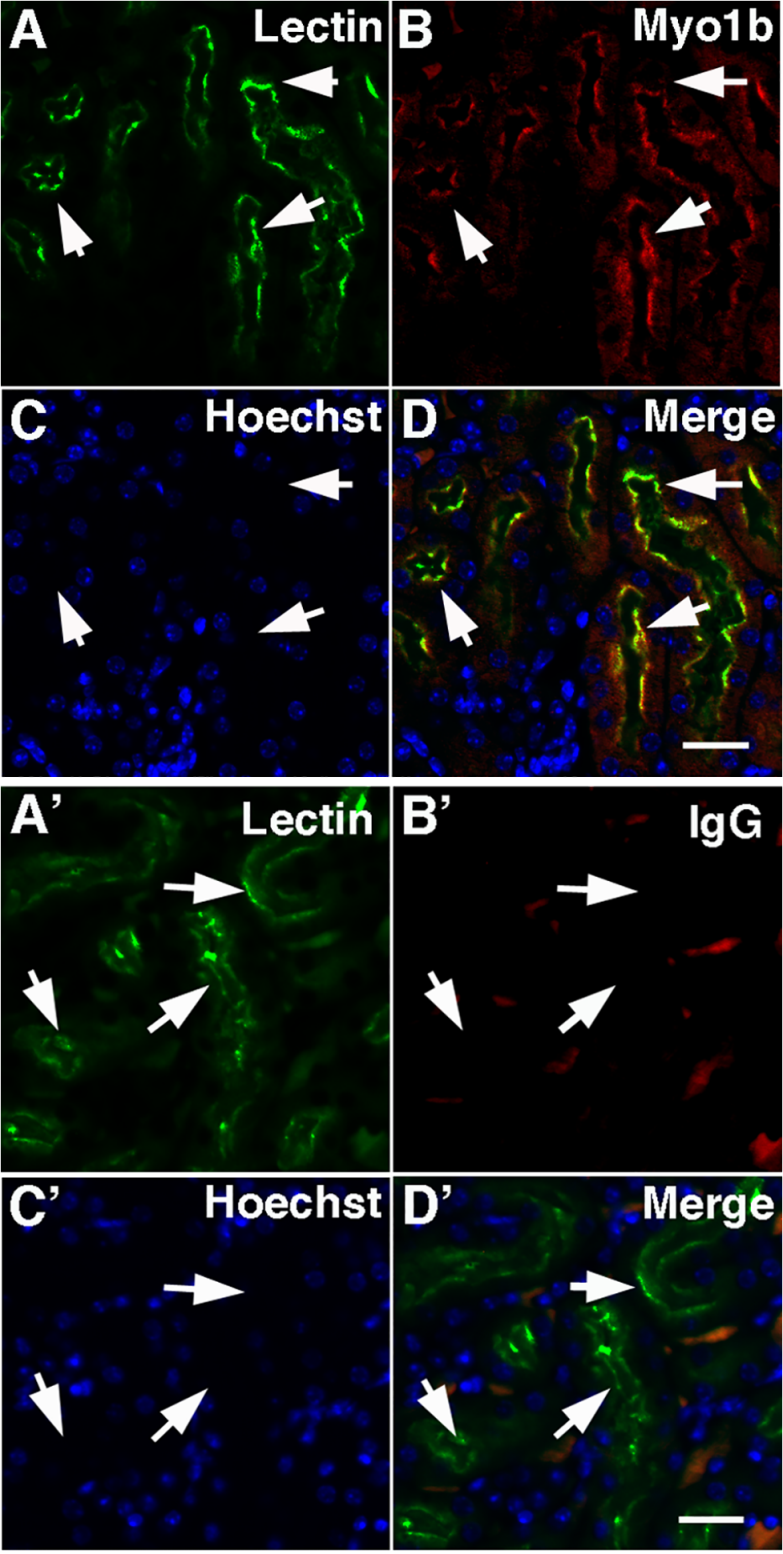
In mouse kidney Myo1b is found in PT cells. In kidney cross-sections treated with Hoechst stain to identify nuclei (*C*, *blue*), Myo1b stained with anti-Myo1b antibodies (B, *red*) co-localized with *Lotus tetragonolobus* lectin, a marker for PTs (A, *green*). D is a merged image of A-C. The arrows identify the corresponding region in each panel. *Scale bar* = 20 μm. A’-D’ are control images of sections stained with lectin (A’), isotype antibodies (B’), and Hoechst (C’); D’ is a merge of A’-C’. The arrows, which identify PTs in A’, point to the corresponding location in each panel. *Scale bar* = 20 μm.

**Fig 2:**
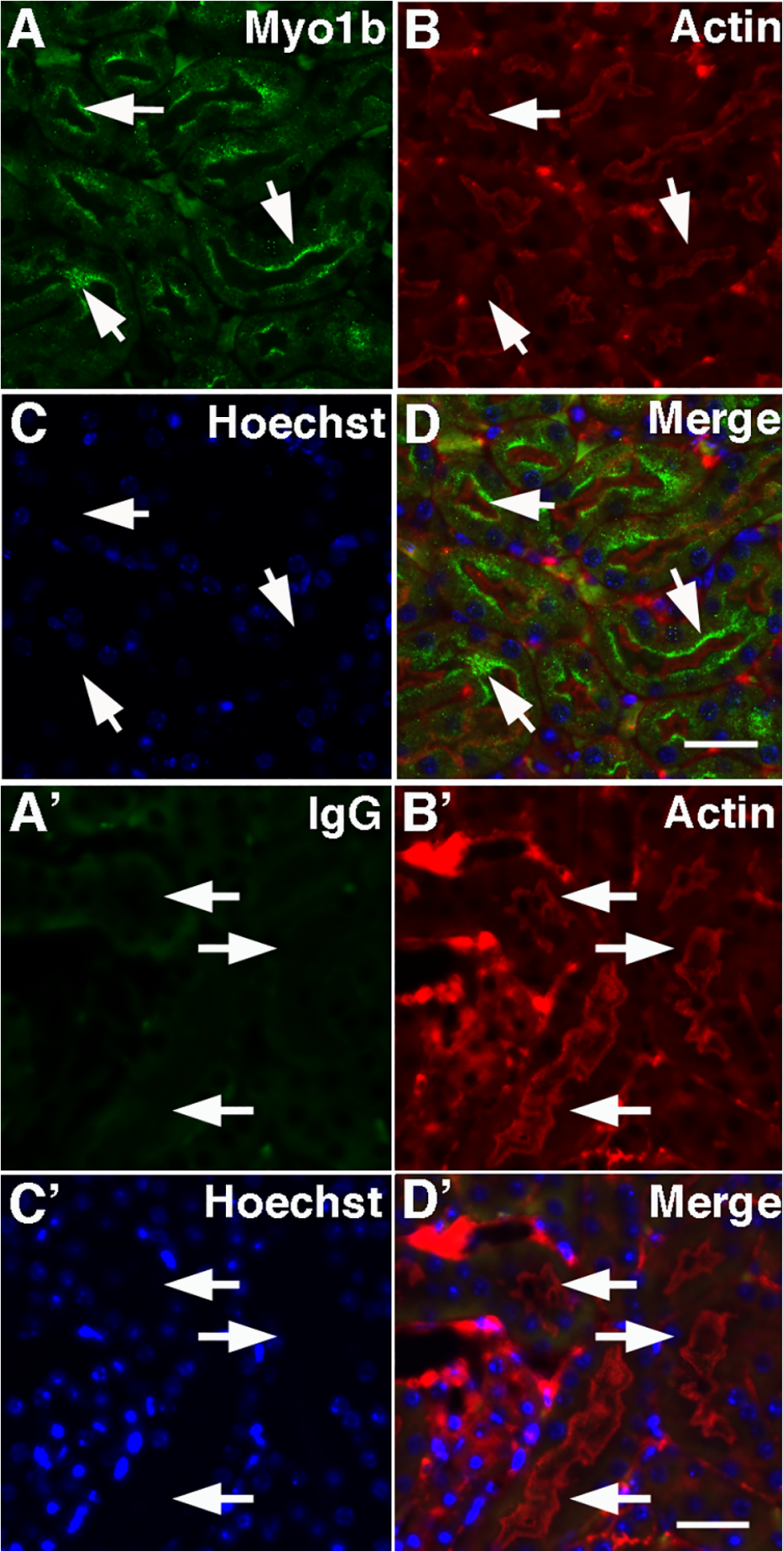
Myo1b localizes to the brush border of renal PT cells. In mouse kidney cross-sections, Myo1b (A, *green*) identified with anti-Myo1b antibodies was at the brush border of PT cells identified by staining of apical microvilli with anti-actin antibodies (B, *red*). *C*, Hoechst stain. D, Merge. *Scale bar* = 20 μm. A’–D’ are control images of renal PT cells using rabbit IgG (A’). *B*’ is stained with anti-actin antibodies. C’ is stained with Hoechst, and D’ is a merged image. The arrows point to the corresponding location in each image. *Scale bar* = 20 μm.

In paraffin sections of mouse kidney cortex, Myo1b (*red*) localized by immunocytochemistry to PTs identified with *Lotus tetragonolobus* lectin (*green*) (Fig 1). Control sections incubated with rabbit IgG and anti-rabbit IgG-Alexa 594 showed no specific staining in the red channel (B’). Myo1b staining (*green*) of kidney cortex was coincident to staining with anti-actin antibodies used to identify the actin-rich microvilli (*red*) comprising the apical brush border of PT cells (Fig 2).

These results placed Myo1b at the brush border, the region of the kidney known to be involved in AAT, and led us to further studies using cultured OK 3B/2 cells to investigate whether Myo1b participates in AAT. OK 3B/2 cells, which derive from opossum (*Didelphys virginiana)* kidney, retain an epithelial cell-like morphology [48,51] with patched microvilli [50,52,53] and exhibit AAT consistent with that of PTs [12,51,54–60]. They express several AATers including collectrin-dependent B^0^AT1 and SIT1 [12,54–56].

**Fig 3:**
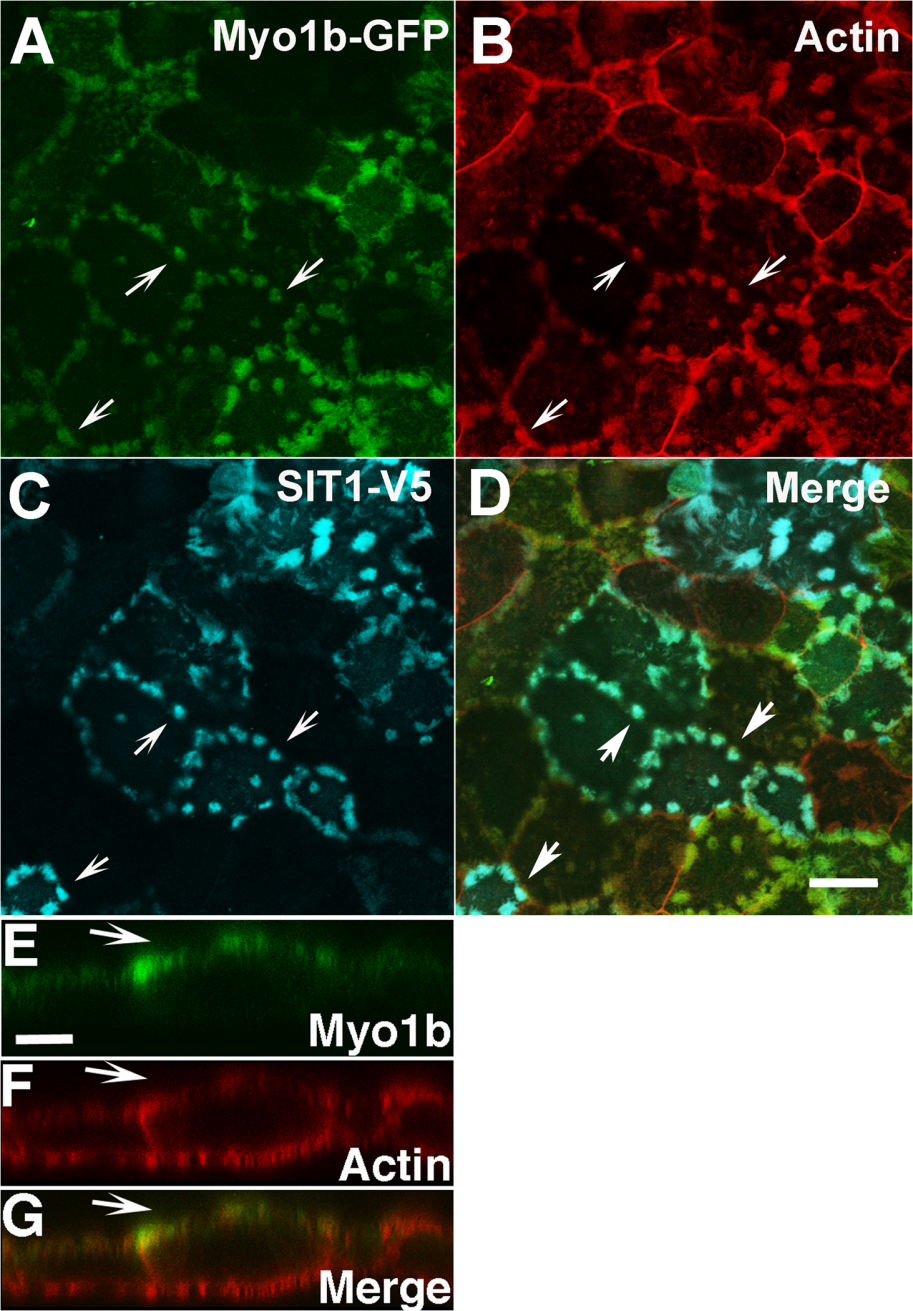
Expressed tagged rat Myo1b localized with the AATer SIT1 in apical microvilli of OK 3B/2 cells. Myo1b-GFP (A, *green*) was found in patched microvilli, which are often located at the cell periphery of OK 3B/2 cells. The actin in the microvilli was identified by rhodamine phalloidin (B, *red*). SIT1-V5 (C, *blue*) localized to the apical microvilli with Myo1b. D is a merged image of *A*—*C*. Arrows identify actin-rich patched microvilli with both Myo1b-GFP and SIT1-V5. A-D are *x-y* images. *Scale bar*, A-D = 20 μm. In *x-z* sections (E-G), Myo1b-GFP (E, *green)* localized in OK 3B/2 cells primarily in actin-containing microvilli (F, *red*) on the apical plasma membrane. *G* is a merge of *E* and *F*, where yellow represents regions of overlap; the arrows point to the position of the apical microvilli in *E*-*G*. *Scale bar*, *panels* E-G = 10 μm.

Studies to localize endogenous Myo1b in OK cells by immunocytochemistry failed even when applying the same antigen retrieval approach that was successful in mouse kidney. This could be due to damage to the microvilli on the surface of OK cells by the antigen retrieval method; phalloidin staining of microvilli is also lower after antigen retrieval methods. Alternatively, this might be because the antigen remains masked in OK cells. Therefore, we expressed and imaged GFP-tagged Myo1b in OK cells. The C-terminal GFP tag does not affect the localization of Myo1b as both endogenous and expressed Myo1b-GFP localize at the periphery of cultured normal rat kidney (NRK) cells [61]. Myo1b-GFP localized to actin-rich microvilli, which typically form patches on the APM of OK 3B/2 cells often at the cell periphery as observed with rhodamine-phalloidin staining (Fig 3A and 3B).

**Fig 4:**
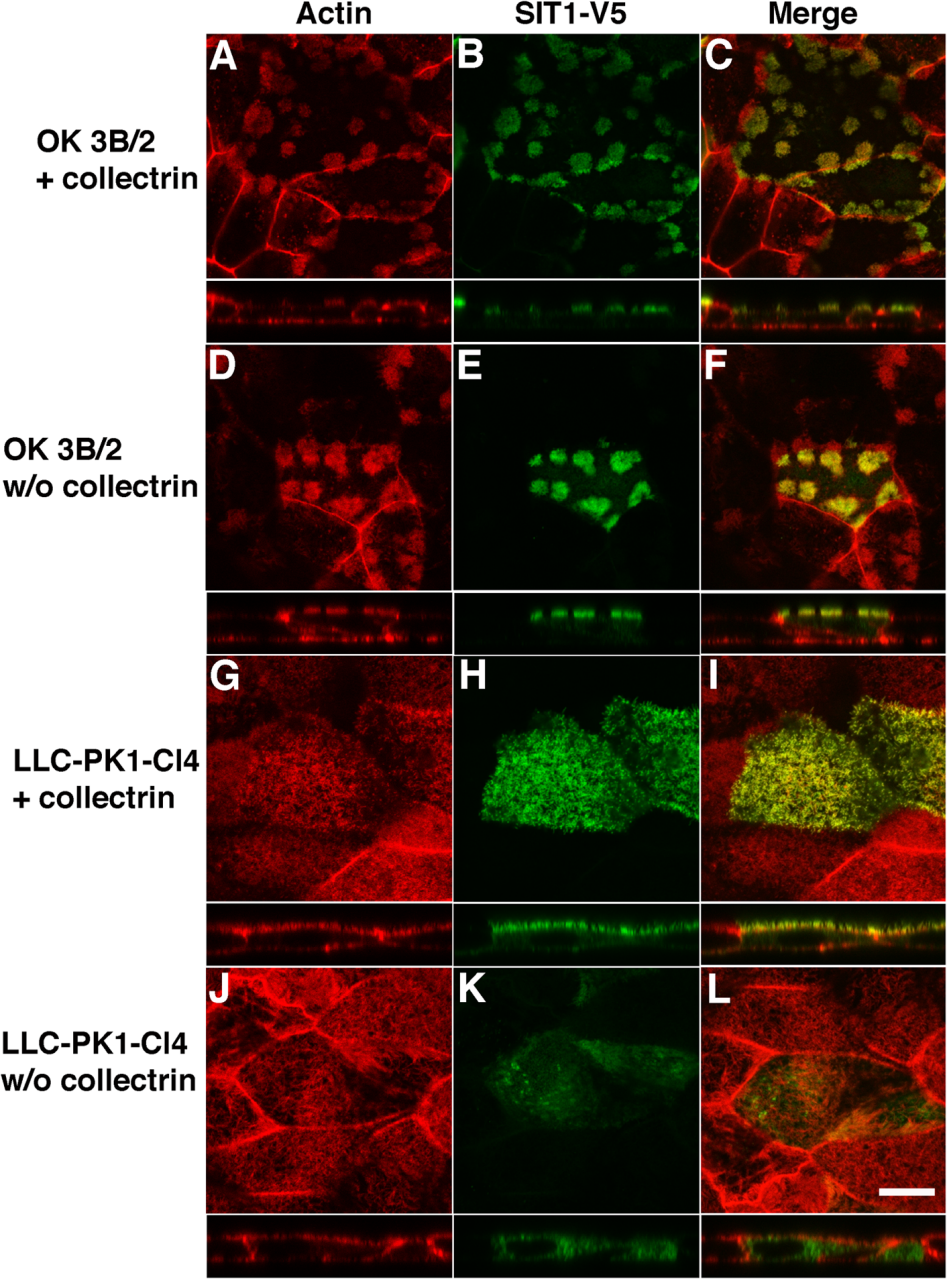
SIT1-V5 localization in microvilli requires co-expression with exogenous collectrin in LLC-PK1-Cl4 cells, but not OK 3B/2 cells. OK 3B/2 cells (A-F) and LLC-PK1-Cl4 cells (G-L) were transiently transfected with SIT1-V5 (*green*) and (+) exogenous collectrin (*A-C* and G-I) or no (w/o) collectrin (D-F and J-L), and stained with rhodamine phalloidin (*red*) to image the apical microvilli (A, D, G, and J). SIT1-V5 localized in patched actin-rich microvilli on the apical surface of OK 3B/2 cells expressing or not expressing exogenous collectrin (A and B; D and E). In LLC-PK1-Cl4 cells, SIT1-V5 localized to microvilli distributed on the apical membrane when co-expressed with collectrin (G-I); however, in the absence of exogenous collectrin, SIT1-V5 was cytoplasmic (J). Merged images (*yellow*) are shown in C, F, I, and L for A and B, D and E, G and H, and J and K, respectively. For each panel, the *x-y* image is shown with the *x-z* image below. *Scale bar* in panel L = 10 μm and applies to all panels.

B^0^AT1 is the major neutral AATer in kidney [4,62,63], but in OK cells the imino acid transporter SIT1 is a major neutral AATer [12]. Because no antibody against OK SIT1 was commercially available, we expressed and localized exogenous mouse SIT1-V5. Like rat Myo1b-GFP, SIT1-V5 localized to the patched microvilli found at the APM in OK 3B/2 cells (Fig 3C). Myo1b-GFP localized in OK 3B/2 cells to the patched microvilli at the APM regardless of whether collectrin was co-expressed (Fig 4A–4C and 4D–4F). In contrast, careful inspection of microvilli that cover the apical membrane of the pig proximal tubule cell line LLC-PK1-Cl4 [64,65] showed that they were positive for SIT1-V5 only when exogenous collectrin was co-expressed (Fig 4G–4I). In LLC-PK1-Cl4 cells not expressing exogenous collectrin, SIT1-V5 was cytoplasmic (Fig 4J–4L); this is most evident in the *x-z* sections.

### RNAi treatment reduced Myo1b expression

**Fig 5:**
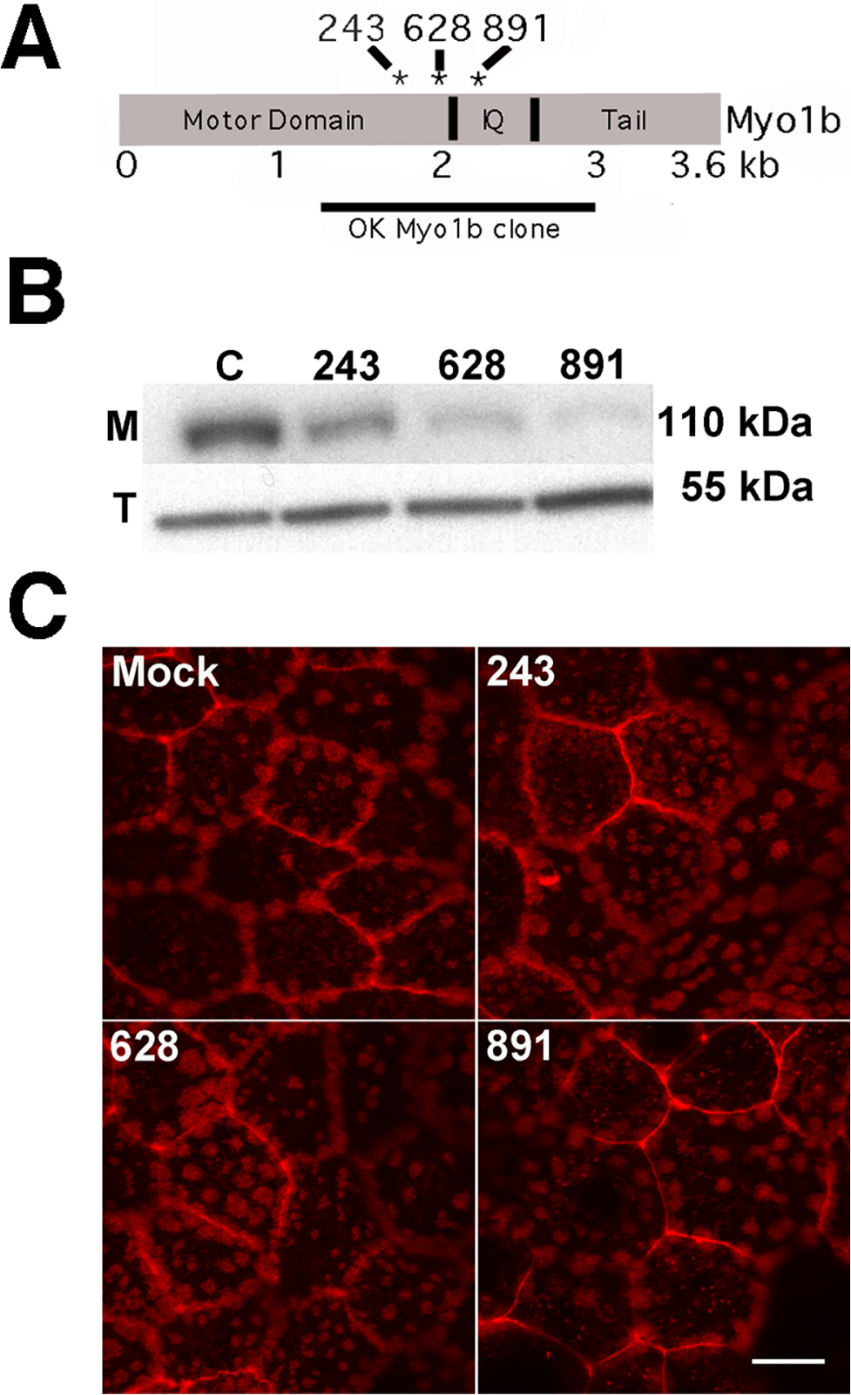
Myo1b kd does not alter microvillar integrity. *A*, Diagram showing the regions in the Myo1b molecule targeted by shRNAs 242, 628, and 891. The region of the Myo1b gene cloned from opossum, which includes the C-terminal region of the motor domain, the entire light chain-binding or “IQ” domain, and a portion of the tail region, is indicated. *B*, Immunoblot showing reduced Myo1b expression in OK 3B/2 cells expressing Myo1b-specific shRNAs 243, 628 and 891. Tubulin (*T*) was used as a loading standard. *C*, Phalloidin-stained patched microvilli on the apical surface of cells transfected with empty vector (*Mock*) resembled those on Myo1b-kd cells (*243*, *628*, *891*) indicating that Myo1b kd does not affect microvillar integrity. *Scale bar* = 10 μm.

To investigate the role of Myo1b in PT cells, we used RNAi to kd Myo1b expression in OK 3B/2 cells. The sequence of *Didelphis virginiana* Myo1b was unknown, so we determined a partial cDNA sequence (GenBank KM588135) in order to design shRNA specific for kd of OK Myo1b (Fig 5A). In the 1662 base pairs cloned, *Didelphis virginiana* Myo1b is 85% identical to the rat Myo1b DNA sequence and 93% identical in amino acid sequence. Lentivirus-mediated expression of shRNA for at least three different sequences (243, 628, 891) resulted in a significant reduction in Myo1b expression (53% for 243; 80% for 628; 99% for 891) as determined by immunoblotting and densitometry (Fig 5B).

### Myo1b kd did not affect the gross structure of microvilli or phosphate transport

**Fig 6:**
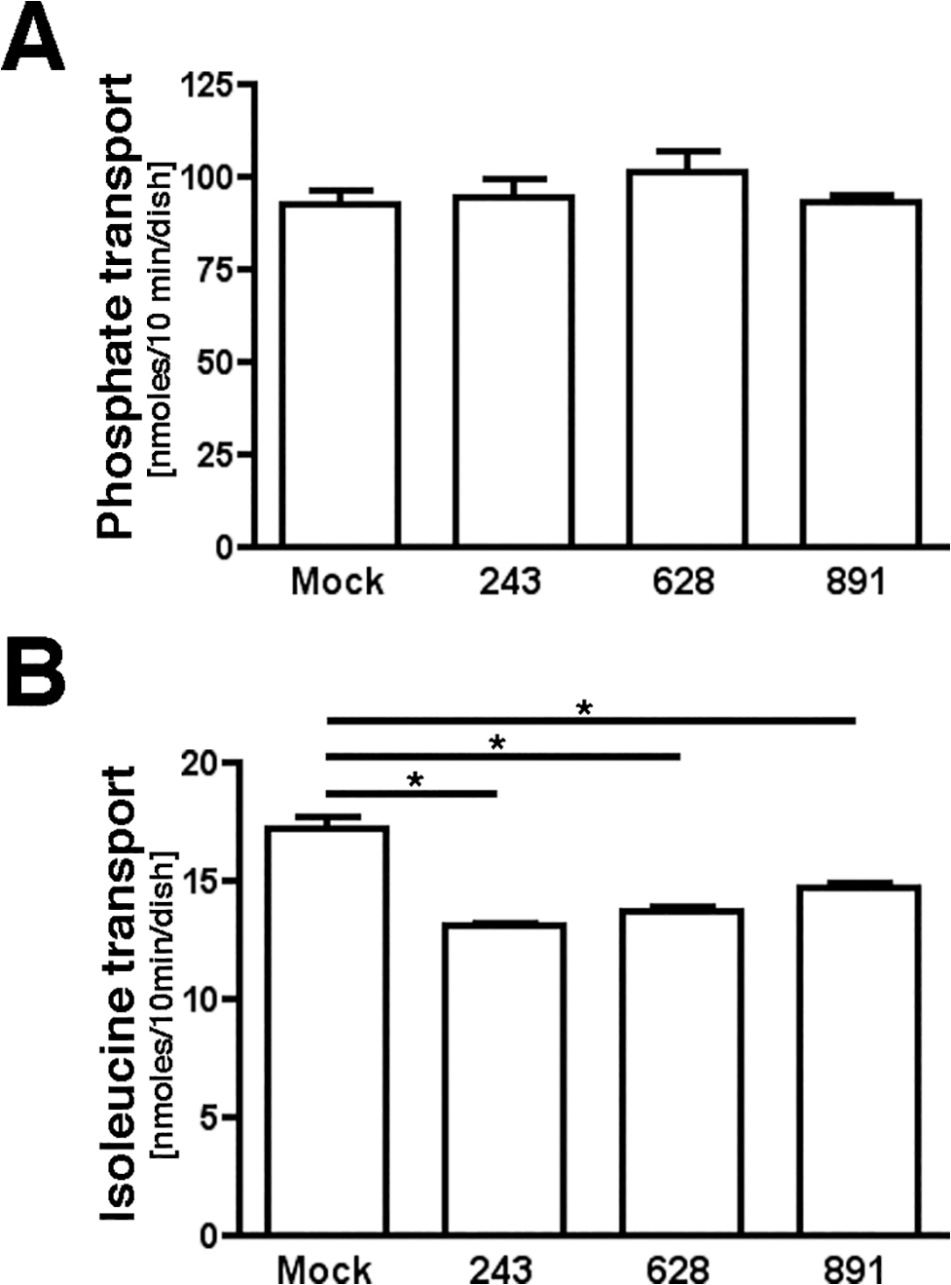
Myo1b kd reduced isoleucine transport, but not phosphate transport in OK 3B/2 cells. A, Phosphate transport was not significantly different in Myo1b-kd vs. control cells when tested at P>0.05. B, OK Myo1b-kd cells (*243*, *628*, and *891*) show reduced [^3^H]isoleucine transport vs. cells transfected with empty vector (*Mock*). Data represent means ± s. d. of samples done in triplicate;*P<0.001.

The effects of Myo1b kd on microvillar structure were determined for evidence that the kd effect was not due to a secondary effect on the structure of microvilli. No obvious changes in the appearance of apical microvilli on the APM were observed by fluorescence microscopy in Myo1b-kd vs. mock-transfected cells stained with rhodamine phalloidin (Fig 5C). This suggested that Myo1b kd did not affect the integrity of the apical microvilli. In addition, Myo1b kd had no effect on [^32^P]phosphate transport (Fig 6A). [^32^P]phosphate transport is mediated by the sodium/phosphate co-transporter NaPi2a, which localizes to microvilli of PT cells [66] and is expressed in OK cells [49]. The studies are evidence that Myo1b kd did not affect all classes of transporters.

### Myo1b kd resulted in reduced neutral amino acid transport

Isoleucine is efficiently transported in OK 3B/2 cells, and SIT1-kd results in reduced isoleucine transport [12]. Using radioactive AAT assays, we found that [^3^H]isoleucine transport was significantly reduced in each of the three pools of Myo1b-kd cells (243, 628, 891) as compared to control (*Mock*) cells (Fig 6B). Reduced AAT by Myo1b kd was not limited to isoleucine; a similar reduction in [^3^H]proline,-alanine, and-leucine transport was observed in Myo1b-kd cells (data not shown).

### OK cells expressing reduced amounts of Myo1b have less SIT1-V5 on the cell surface

**Fig 7:**
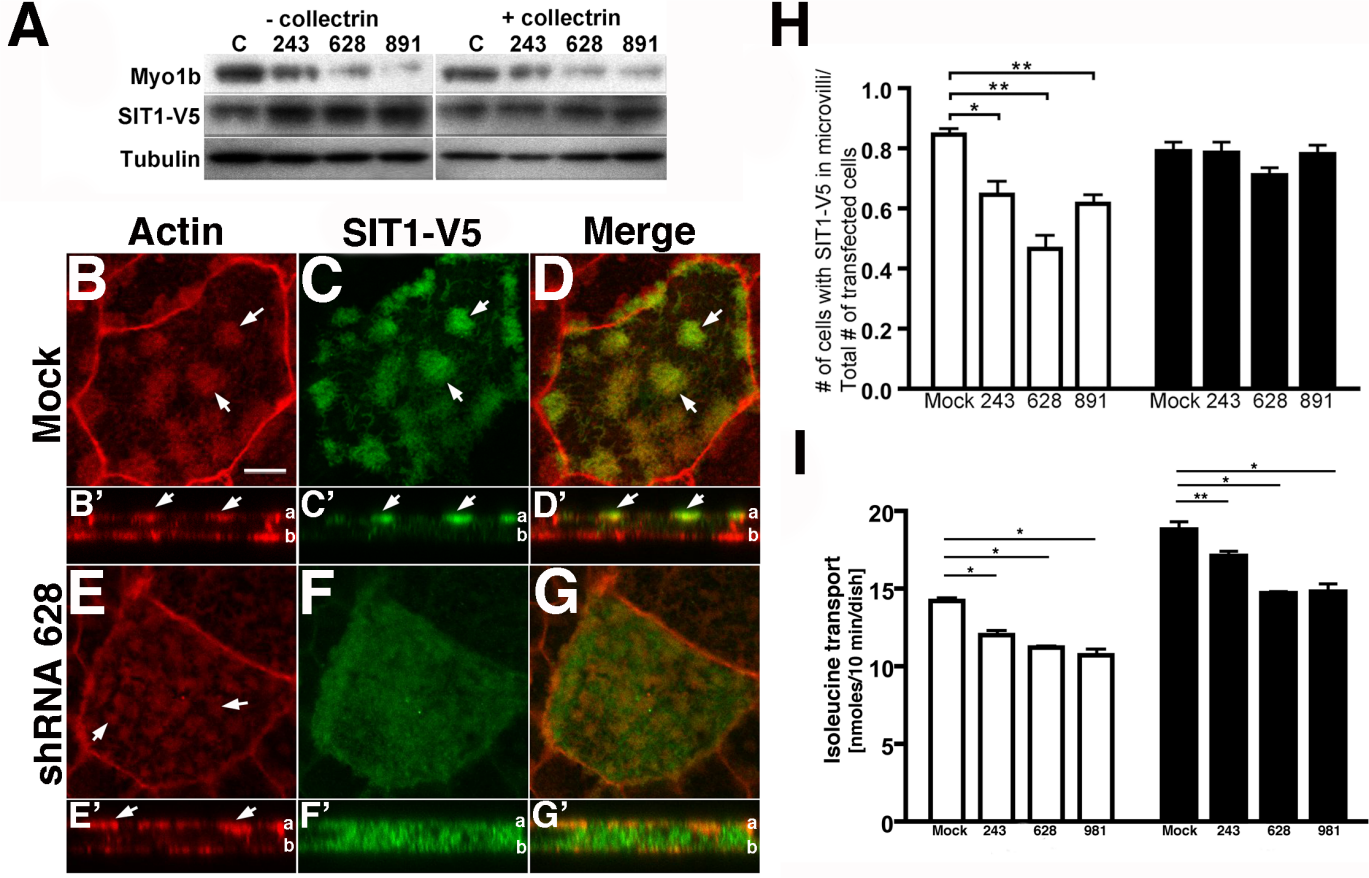
Myo1b kd reduces the number of SIT1 transporters at the apical plasma membrane. OK cells were transfected with SIT1-V5 and either collectrin or empty vector (pLenti-CMV Hygro) and stained with rhodamine phalloidin to visualize the apical microvilli. A, Immunoblot showing that Myo1b kd increased SIT1-V5 expression. Tubulin served as a loading control. *B-G*, B’-G’, Sample images showing that in control (Mock) OK 3B/2 cells (B-D; B’-D’), expressed SIT1-V5 (C, *green*) localized almost exclusively in actin-rich microvilli (B, *red; arrows*) on the apical surface; whereas in Myo1b kd-cells (shRNA 628) (E-G; E’-G’), SIT1-V5 localized to the cytoplasm. Cytoplasmic SIT1-V5 in Myo1b-kd cells is most evident in the lateral (*x-z*) view shown in F’. B-G are *x-y* images; B’-G’ are *x-z* images of B-G, respectively. In B’-G’, the position of the apical membrane is marked with *a*, and the position of the basal membrane is marked with *b*. D, D’, G, G’, Merge, *yellow*. *Bar*, 5 μm. H, Myo1b-kd decreased the number of cells expressing SIT1-V5 at the APM. The number of transfected cells with SIT1-V5 in apical microvilli was determined and expressed as a function of the total number of transfected cells. In each case 167–465 transfected cells were counted. The data are from three independent experiments and expressed as means ± s.e. *P<0.01, ** P<0.005. The expression of exogenous collectrin rescued the Myo1b-kd-induced decrease in SIT1-V5 at the APM. The number of cells expressing SIT1-V5 at the APM was not statistically different in control vs. Myo1b-kd cells when collectrin was overexpressed; P>0.05. I, [^3^H]isoleucine transport was not rescued in Myo1b-kd cells by collectrin. Overexpression of exogenous collectrin in Myo1b-kd cells did not rescue neutral amino acid transport. White filled bars, (-) collectrin; black-filled bars, (+) collectrin. Data are means ± s. d. for samples done in triplicate. * P<0.005, ** P<0.001.

We considered the possibility that the reduced AAT observed in Myo1b-kd cells was a consequence of fewer transporters at the APM. We tested this hypothesis with OK 3B/2 cells expressing mouse SIT1-V5 and either Myo1b-specific shRNA or empty vector. First, we investigated by immunoblotting whether Myo1b kd affects expression of SIT1-V5. We found that Myo1b kd modestly increases the amount of SIT1-V5 protein in whole cell extracts especially in Myo1b-kd cells not expressing exogenous collectrin (Fig 7A).

Then, we examined control and Myo1b-kd cells stained for actin and SIT1-V5 (Fig 7B–7G’). We found in *x-z* sections of control cells that SIT1-V5 localized exclusively at the APM (C, C’); whereas in Myo1b-kd cells SIT1-V5 was often cytoplasmic (F, F’). Analysis by immunofluorescence microscopy showed that expression of Myo1b-specific shRNA resulted in 25–45% fewer cells with SIT1-V5 in apical microvilli vs. the cytoplasm than cells treated with empty vector (Fig 7H).

Interestingly, expression of exogenous collectrin rescued the decrease in the amount of SIT1-V5 at the APM in Myo1b-kd cells suggesting a role of Myo1b in the association of collectrin-dependent AATers with the APM (Fig 7H). However, although collectrin expression increased the amount of [^3^H]isoleucine transport in both control and Myo1b-kd cells, [^3^H]isoleucine transport by Myo1b-kd cells was still less than observed in control cells, suggesting that although collectrin expression resulted in more SIT1-V5 at the APM, it did not reverse the effect of Myo1b kd on AAT (Fig 7I).

## Discussion

In OK 3B/2 cells SIT1 is the major neutral AATer [12]. The localization of Myo1b at the renal brush border coupled with the observed decrease in the amount of SIT1 at the APM in OK cells and reduction in [^3^H]isoleucine transport following kd of Myo1b expression suggest a model in which Myo1b facilitates AAT by supporting the association of AATers with the microvilli-rich APM of renal PT cells. Interestingly, a similar role has recently been ascribed to collectrin, a homolog of ACE2. Collectrin-null mice show reduced amounts of several AATers, including B^0^AT1 and SIT1, at the brush border membrane of renal PT cells and severe aminoaciduria [15,16]. We expected that expression of exogenous SIT1-V5 would require co-expression with collectrin based on studies showing that transport activity of B^0^AT1 expressed in MDCK cells requires collectrin [15] and our studies in LLC-PK1-Cl4 cells showing that localization of SIT1-V5 at the APM required collectrin (Fig 4). However, this was not the case, and exogenous SIT1-V5 expression and its localization to the APM did not require exogenous collectrin in OK 3B/2 cells (Fig 4). It is possible that OK 3B/2 cells express higher levels of endogenous collectrin than MDCK or LLC-PK1-Cl4 cells.

Expression of exogenous collectrin rescued the decrease in SIT1-V5 observed at the APM in Myo1b-kd cells (Fig 7C). Compensation by collectrin suggests that collectrin and Myo1b participate in the same process. However, although [^3^H]isoleucine transport increased in both control and Myo1b-kd cells expressing exogenous collectrin, the amount of [^3^H]isoleucine transport in Myo1b-kd cells expressing exogenous collectrin was still less than that of controls. That collectrin expression increases the number of AATers at the APM of Myo1b-kd cells, but not AAT, suggests that without Myo1b the AATers may not be properly inserted into the membrane, and hence, are nonfunctional. Although we can successfully biotinylate SIT1-V5 in LLC-PK1-Cl4 cells, we have been unable to use cell surface biotinylation assays in OK cells to determine whether (i) the amount of properly exocytosed SIT1-V5 is reduced by Myo1b kd, and (ii) the SIT1-V5 appearing at the membrane in OK Myo1b-kd cells expressing exogenous collectrin is actually inserted into the membrane. One possibility is that Myo1b supports exocytosis of SIT1 by mediating membrane fusion of vesicles containing SIT1. Membrane binding of Myo1b [33] and its exquisite sensitivity to force [40] suggest that Myo1b contributes to the force required for membrane fusion. Alternatively, Myo1b mediates trafficking of AATers to the APM and/or tethers AATers at the APM so that they function properly.

Collectrin knockout causes a reduction in protein expression of collectrin-dependent AATers including SIT1 [15]; however, we found no reduction in SIT1-V5 expression in Myo1b-kd cells whether or not exogenous collectrin was coexpressed. In fact, we noted a small increase in SIT1-V5 expression especially in OK Myo1b-kd cells not expressing exogenous collectrin (Fig 7A). This increase might be a compensatory mechanism. Most importantly, the localization of SIT1-V5 shifted from the apical microvilli to the cytoplasm. The accumulation of SIT1-V5 in the cytoplasm of Myo1b-kd cells (Fig 7H) is evidence that Myo1b is necessary for the association of SIT1-V5 with the APM perhaps by supporting fusion of SIT1-V5-containing vesicles, or alternatively, tethering SIT1-V5 once it is inserted into the membrane.

How Myo1b associates with SIT1 is unknown. Attempts to co-immunoprecipitate myc-Myo1b and SIT1-V5 with anti-myc or anti-V5 antibodies failed. Several reasons can account for this result. For one, SIT1 is a membrane protein, and conditions that extract SIT1 from the membrane are likely also to disrupt the interaction between SIT1 and its possible binding partner Myo1b. The extraction conditions might be particularly critical if Myo1b binds indirectly to SIT1 such that multiple interactions must remain intact for successful immunoprecipitation. In addition, the interaction of Myo1b with SIT1 might be direct, but weak or transient.

The effect of Myo1b on collectrin-dependent AATers suggests a relationship among AATers, collectrin and Myo1b. Collectrin colocalizes with several different AATers at the brush border suggesting that it forms a complex with them; however, whether collectrin binds directly to AATers or interacts with AATers through a linker protein is unknown. Our work suggests that Myo1b may be another component of AATer-collectrin complexes; however, confirmation of this model and the nature of the putative AATer-collectrin-Myo1b complex await further investigation. Collectrin associates with SNARE proteins [17,18], which mediate vesicle fusion and intracellular trafficking. Curiously, Myo1b associates with several intracellular compartments [32,34] and is also implicated in intracellular trafficking [36,37,43]. It is possible that Myo1b modulates the fusion of collectrin-dependent AATers with the APM, a role previously reported for Myo1c and GLUT4-containing vesicles at the adipocyte plasma membrane [67]. Myo1b and AATers may be trafficked together following their synthesis in the ER; however, when Myo1b first associates with AATers has not yet been determined.

Myo1b kd does not eliminate all isoleucine transport. This could be due to the presence of collectrin-*in*dependent neutral AATers in OK 3B/2 cells. In addition, Myo1b kd could provoke increased expression of collectrin-*in*dependent transporters. Expression of some AATers increases in collectrin-knockout mice [16], compensating for the loss of collectrin. Alternatively, small amounts of Myo1b remaining after RNAi treatment could be responsible for the remaining AAT. In addition, other class I myosins could compensate for Myo1b in OK 3B/2 Myo1b-kd cells. For example, Myo1d redistributes along the length of intestinal microvilli in Myo1a-null mice[68]. Myo1c and Myo1d are found in renal brush borders of PTs by proteomic analyses [69], and all class I myosins are identified in kidney by transcriptomic analyses [70,71].

The amino-terminal motor domain of class I myosins contains nucleotide- and actin-binding sites, whereas the carboxyl-terminal tail domain contains sites for membrane binding and presumably binding sites for other molecules termed “cargo”. We previously determined that Myo1b binds PIP_2_ and PIP_3_ specifically and with high affinity through a putative PH domain in the tail domain [33]. The tail region is critical to the proper localization of Myo1b in protrusions at the plasma membrane, and instead of localizing to membrane protrusions, Myo1b mutant in the putative PH domain is cytoplasmic [33]. One idea is that Myo1b associates indirectly with AATers to tether them to the APM through its membrane-binding site. A role for Myo1b in tethering AATers to the APM is consistent with that previously proposed for class I myosins. In particular, Myo1a anchors sucrase-isomaltase [28] and possibly CFTR channels [30] in the intestinal microvillar membrane; Myo1c tethers GLUT4-containing vesicles in muscle cells [72] and mediates their fusion with the adipocyte plasma membrane [67,73]; and recent results from our laboratory show that Myo1c stabilizes E-cadherin at sites of cell-cell contact in polarized MDCK epithelial cells [74]. Together, the studies support the idea that class I myosins tether membrane-associated proteins to the actin cytoskeleton. Myo1b is widely expressed [31], and collectrin has been found in a few different tissues [14,75,76], so it is possible that the interplay of Myo1b and collectrin affects other cellular processes.

The studies reveal a new physiological role for Myo1b in renal microvilli, that of supporting AAT by tethering AATers to the APM. In intestine Myo1a regulates polarization and differentiation of colorectal cancer cells, is mutated in colorectal cancers, and has tumor-suppressor activity [77] suggesting that equally important physiological roles for Myo1b in humans will ultimately be found.
